# A Pyrrole Modified 3,4‐Propylenedioxythiophene Conjugated Polymer as Hole Transport Layer for Efficient and Stable Perovskite Solar Cells

**DOI:** 10.1002/smll.202408440

**Published:** 2024-10-28

**Authors:** Yuanhao Tang, Ke Ma, Wenhao Shao, Yoon Ho Lee, Ashkan Abtahi, Jiaonan Sun, Hanjun Yang, Aidan H. Coffey, Harindi Atapattu, Mustafa Ahmed, Qixuan Hu, Wenzhan Xu, Raunak Dani, Limei Wang, Chenhui Zhu, Kenneth R. Graham, Jianguo Mei, Letian Dou

**Affiliations:** ^1^ Davidson School of Chemical Engineering Purdue University West Lafayette IN 47907 USA; ^2^ Global Institute of Future Technology Shanghai Jiao Tong University Shanghai 200240 China; ^3^ Department of Chemistry Purdue University West Lafayette IN 47907 USA; ^4^ Advanced Light Source Lawrence Berkeley National Laboratory Berkeley CA 94720 USA; ^5^ Department of Chemistry University of Kentucky Lexington KY 40506 USA; ^6^ Birck Nanotechnology Center Purdue University West Lafayette IN 47907 USA

**Keywords:** hole transport layer, PEDOT, perovskite solar cells, ProDOT, pyrrole

## Abstract

Despite the outstanding electric properties and cost‐effectiveness of poly(3,4‐ethylenedioxythiophene) (PEDOT) and its derivatives, their performance as hole transport layer (HTL) materials in conventional perovskite solar cells (PSCs) has lagged behind that of widely used spirobifluorene‐based molecules or poly(triaryl amine). This gap is mainly from their poor solubility and energy alignment mismatch. In this work, the design and synthesis of a pyrrole‐modified HTL (PPr) based on 3,4‐propylenedioxythiophene (ProDOT) are presented for efficient and stable PSCs. As a result of the superior defects passivation ability, excellent contact with perovskite, enhanced hole extraction, and high hydrophobicity, the unencapsulated PPr‐based PSCs showed the peak PCE of 21.49% and outstanding moisture stability (over 4000 h). This work highlights the potential application of ProDOT‐based materials as HTL for PSCs and underscores the importance of the rational design of PEDOT and its derivatives.

## Graphical abstract

A pyrrole unit is introduced into the backbone of 3,4‐propylenedioxythiophene (PPr) and copolymerized as the hole transport layer materials for perovskite solar cells (PSCs). As a result of the superior defects passivation ability, excellent contact with perovskite, enhanced hole extraction, and high hydrophobicity, the unencapsulated PPr‐based PSCs showed an impressive peak power conversion efficiency of 21.49% and outstanding moisture stability (over 4000 h).

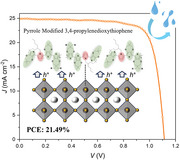

## 1 Introduction

Perovskite solar cells (PSCs) have attracted considerable attention due to their exceptional power conversion efficiency (PCE) and the potential for cost‐effective manufacturing.^[^ ^1^ ^]^ The hole transport layer (HTL) within PSCs plays a key role in facilitating efficient hole transport and suppressing undesired reverse electron transfer.^[^ ^2^ ^]^ Currently, 2,2,7,7′‐tetrakis (N, N‐di‐p‐methoxyphenylamine)−9,9′‐spirobifluorene (Spiro) and poly(triaryl amine) (PTAA) are the dominant HTL materials used in conventional n‐i‐p structured PSCs due to their superior device performance.^[^ ^3^ ^]^ However, Spiro suffers from thermal degradation and inadequate energy barriers for ion migration, leading to significant stability issues.^[^ ^4^ ^]^ PTAA, although thermally stable, requires precise control over molecular weight and the exclusion of trace metal impurities, which substantially increases its cost.^[^ ^5^ ^]^ Moreover, PTAA is prone to moisture ingress, causing the decomposition of perovskite (PVSK) and delamination at the PVSK‐HTL interface.^[^ ^6^ ^]^ Thus, it is imperative to delve into the potential of alternative HTL materials.

Poly(3,4‐ethylenedioxythiophene) (PEDOT) has been utilized as an HTL in both conventional and inverted photovoltaics due to its low cost, ease of processing, and chemical stability.^[^ ^7^ ^]^ However, the performance of PEDOT‐based materials, primarily PEDOT: poly(styrene sulfonate) (PEDOT:PSS), has been far less than satisfactory. This is mainly due to the significant energy level mismatch between PVSK and PEDOT:PSS, which leads to inefficient charge extraction and potential energy losses.^[^ ^8^ ^]^ Additionally, the electrochemical properties of PEDOT are highly dependent on the electro‐polymerization process involving specific dispersants, making it challenging to finely tune the electronic structure.^[^ ^9^ ^]^ Earlier researchers tried to increase the work function of PEDOT:PSS by incorporating the polymer electrolyte^[^ ^10^ ^]^ or inserting an ultrathin PTAA layer between PEDOT:PSS and PVSK.^[^ ^11^ ^]^ Despite various efforts, the PCE of PEDOT‐derived HTLs continues to fall short of expected levels, with the possibility of instability issues arising from the incorporation of extra additives or interlayers.

To improve the synthetic flexibility, 3,4‐propylenedioxythiophene (ProDOT) with two reactive hydrogen positions on the side chain was developed.^[^ ^12^ ^]^ A series of high‐solubility and conductive copolymers combining ProDOT with 3,4‐ethylenedioxythiophene (EDOT) were demonstrated, in which their energy levels can be easily tuned by changing the ratio of ProDOT to EDOT.^[^ ^13^ ^]^ Nevertheless, the energy level alignment of the poly(ProDOT*_x_*−EDOT*_y_*) (PxEy) series remains a challenge, as their shallow highest occupied molecular orbital (HOMO) may introduce trap states at the PVSK‐HTL interface, thereby reducing charge transfer efficiency.

Herein, a pyrrole‐modified dioxythiophene copolymer (denoted as PPr) was successfully developed and applied as the HTL in PSCs. The introduction of the pyrrole in PPr significantly decreased the energy mismatch with PVSK by twisting the polymer backbone, thus lowering the HOMO energy, and enhancing the hole extraction capabilities. Consequently, an outstanding PCE of 21.49% with an enhanced open‐circuit (*V*_oc_) of 1.11 V, a fill factor (FF) of 77.5%, and a short circuit (*J*_sc_) of 24.89 mA cm^−2^ has been achieved. Furthermore, the uniform coverage and successful passivation from PPr lead to the excellent stability of the devices. PPr‐based devices exhibited exceptional humidity stability, maintaining 94% of their initial PCE after aging in the air for more than 4000 h. Additionally, PPr‐based devices exhibited remarkable light stability (80% after 1400 h) and thermal stability (76% at 65 °C for 1800 h). This study demonstrates the application of PPr as the HTL for PSCs and provides a foundation for further investigation and optimization of PEDOT and its derivatives.

## 2 Results and Discussion

**Figure 1:**
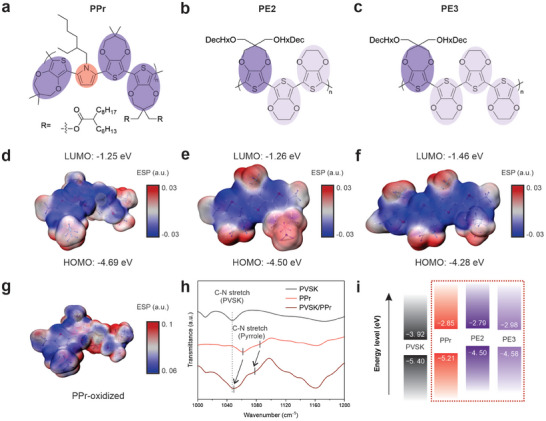
Molecular structures of a) PPr, b) PE2, and c) PE3. ESP maps and DFT calculated energy levels of d) PPr, e) PE2, f) PE3, and g) oxidized PPr. h) The FT‐IR spectra of PVSK, PPr, and PVSK/PPr. i) The energy diagram of PVSK, PPr, PE2, and PE3 based on UV–vis and UPS results.

We synthesized a ProDOT‐Pyrrole‐ProDOT trimer and subsequently polymerized it with an ProDOT monomer to get PPr (**Figure** **1**a; Figures S1–S7, Supporting Information). Due to the high conductivity and accessibility, poly(ProDOT−EDOT_2_) (PE2) and poly(ProDOT−EDOT_3_) (PE3) were added as comparison HTL materials (Figure 1b,c). Density functional theory (DFT) results showed that the introduction of pyrrole deepened the HOMO energy of the corresponding monomer from −4.50 eV (PE2), and −4.28 eV (PE3) to −4.69 eV (Figure 1d–g; Figure S8, Supporting Information). In addition, the incorporation of pyrrole allowed for the maintenance of large dihedral angles in the neutral state, which, compared to EDOT, suppressed excessive π–π interactions and thus enhanced the solubility (Figure S8, Supporting Information). Interestingly, the dihedral angles decreased from 56° and 45.3° to 30.6° and 22.3° after oxidation, resulting in a relatively planar structure and proper stacking.

The electron‐rich nature of pyrrole makes PPr less susceptible to potential instability from nucleophilic addition and radical‐radical coupling.^[^ ^14^ ^]^ The electrostatic surface potential (ESP) clearly showed that the negative charges were localized around the backbones of all three monomers (Figure 1d–f). Notably, PPr exhibited strong electron enrichment around the pyrrole‐containing unit (Figure 1d). After oxidation, the electron density around the pyrrole ring decreased dramatically compared with the thiophene rings in the backbone (Figure 1g). This easy loss of electrons indicates the reactive nature of the pyrrole unit, thereby underscoring its potential to enhance the doping efficiency of the polymer.^[^ ^15^ ^]^ The lone pair of nitrogen in pyrrole can also promote the formation of Lewis acid‐base adducts, which effectively passivate the uncoordinated lead on the surface of PVSK.^[^ ^16^ ^]^ The Fourier transform infrared spectroscopy (FTIR) spectrum of PPr displayed a typical pyrrole vibration mode, where the peaks at 1085 and 1061 cm^−1^ were assigned to the stretching vibration of the C─N in the pyrrole unit (Figure 1h; Figure S9, Supporting Information).^[^ ^17^ ^]^ It is noted that the intensity of the C─N stretching vibration increased significantly after binding to the surface of the perovskite, indicating strong PVSK‐PPr interaction. The downward shift of the C─N stretching vibration peaks from 1085 to 1078 cm^−1^, and from 1061 to 1050 cm^−1^, respectively, may be attributed to electron delocalization following the formation of Lewis acid‐base adducts.^[^ ^18^ ^]^ Similar to PE2 and PE3, no obvious heat flow peak can be observed in differential scanning calorimetry scans for PPr (Figure S10a, Supporting Information), which indicates the incorporation of pyrrole does not affect the thermostability characteristics of the ProDOT backbone. The thermogravimetric analysis further reveals that the PPr has superior thermostability in comparison to PE2 and PE3, with the melting temperature up to 350 °C (Figure S10b, Supporting Information).

To better understand the energy alignment of polymers, we performed UV–vis absorption and ultraviolet photoelectron spectroscopy (UPS) measurements. The bandgaps of PPr, PE3, and PE2 were determined by the Taut plot and found to be 2.36, 1.6, and 1.7 eV, respectively (Figure S11, Supporting Information). The ionization energies of PPr, PE2, and PE3 were calculated to be −5.21, −4.5, and −4.58 eV, respectively (Figure S12, Supporting Information). The complete energy diagram is shown in Figure 1i, with the detailed results listed in Table S1 (Supporting Information). These findings suggest that the introduction of pyrrole successfully enlarged the bandgap and effectively tailored the HOMO of the ProDOT backbone, thus minimizing the energy level mismatch.

**Figure 2:**
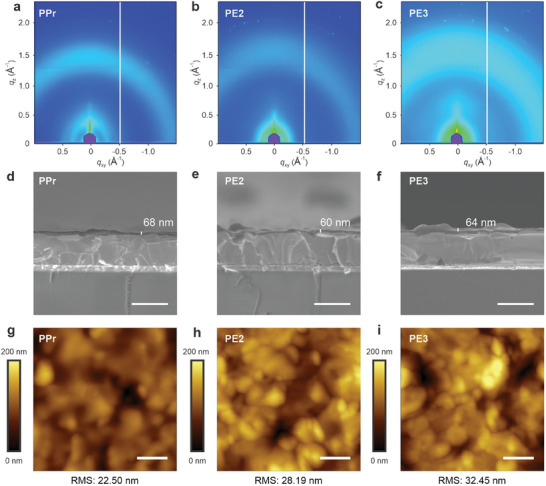
a–c) GIWAXS patterns, d–f) cross‐section SEM images, and g–i) AFM images of PPr (a,d,g), PE2 (b,e,h), and PE3 (g,h,i). The *q*_xy_ and *q*_z_ denote the scattering vectors for in‐plane and out‐of‐plane, respectively. The scale bar is 1 µm for all samples.

The absence of peaks in the powder X‐ray diffraction patterns indicates the amorphous nature of the polymers (Figure S13, Supporting Information). To further gain insights into the crystallinity and intermolecular stack characteristics, grazing incident wide‐angle X‐ray scattering (GIWAXS) was conducted (**Figure** **2**a–c). In GIWAXS profiles, all the polymers exhibit weak diffuse halos, with the intensity of out‐of‐plane line (*q*_z_) cuts being considerably stronger than that of in‐plane line (*q*_*xy*_) cuts (Figure S14, Supporting Information). The observed stacking peak around *q*_z_ ≈ 1.5 Å^−1^ can be attributed to the π–π stacking interaction. Interestingly, PPr demonstrates a narrower π–π scattering diffraction pattern compared with PE2 and PE3, signifying an improved π–π stacking of the PPr.^[^ ^19^ ^]^ The PPr also exhibits pronounced (*h*00) peaks along the *q*_z_ direction, indicating the preferable face‐on orientation (Figure S15, Supporting Information). With enhanced π–π stacking interactions and favorable face‐on orientation, the charge transport and collection processes in PPr‐based devices are expected to improve.

Scanning electron microscopy (SEM) and cross‐sectional SEM were employed to investigate the morphology of different polymers coated on the surface of perovskite. The SEM images revealed well‐covered surface morphologies for all polymers (Figure S16, Supporting Information). Cross‐section SEM showed that all HTLs have similar thicknesses of ≈65 nm (Figure 2d–f). Notably, the PPr layer exhibited superior coverage on the perovskite surface due to its excellent solubility and relatively planar geometry after oxidation. In contrast, the PE3 and PE2 layers showed noticeable grooves when coated on the perovskite layer. Gaps could be observed between the perovskite layer and the PE3 layer, which may hinder effective charge transfer and lead to structure instability (Figure 2f). Atomic force microscopy (AFM) was used to further analyze the top view of HTLs (Figure 2g–i; Figure S17, Supporting Information). The root means square of PPr was calculated to be 22.50 nm on the five‐by‐five µm scale, smaller than the 28.19 and 32.45 nm of PE3 and PE2, respectively (Figure S17, Supporting Information).

**Figure 3:**
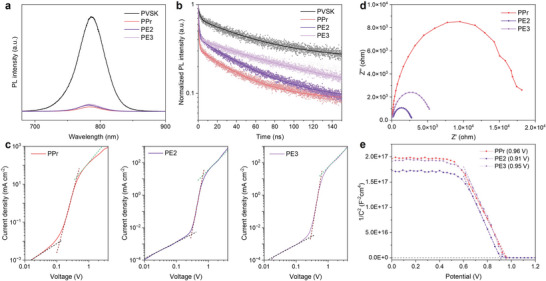
a) PL spectra, and b) TRPL of pristine PVSK and PVSK with PPr, PE2, and PE3 films. c) The SCLC curves, d) Nyquist plots, and e) MS plots of devices based on PPr, PE2, and PE3.

To explore the potential of using these materials as HTLs, we measured the electrical conductivity and hole mobility of PPr, PE2, and PE3. The PPr film exhibited a conductivity of 2.03 × 10^−5^ S·cm^−1^, which is 78.4, and 211.2 times higher than that of PE3 and PE2 films, respectively (Figure S18, Supporting Information). Additionally, the PPr film demonstrated the highest hole mobility, with a value of 1.37 × 10^−4^ cm^2^·V^−1^·s^−1^. In comparison, PE3 film showed a slightly lower mobility of 1.15 × 10^−4^ cm^2^·V^−1^·s^−1^ while PE2 film had the lowest mobility at 0.84 × 10^−4^ cm^2^·V^−1^·s^−1^ (Figure S19, Supporting Information). These results highlight the superior conductivity and hole transport capability of PPr among the evaluated HTL materials. The steady‐state photoluminescence (PL) and time‐resolved PL (TRPL) were conducted to explore the interfacial charge extraction kinetics between PVSK and HTL (**Figure** **3**a,b). The pristine perovskite exhibited a typical PL peak at 786 nm while significant PL quenching was observed in the films coated with HTLs (Figure 3a). PPr displayed the strongest quenching effect compared with PE3 and PE2, indicating the most efficient hole extraction ability. For TRPL, the PPr film showed the fastest *τ*_1_ (hole extraction) of 0.55 ns among all HTLs, whereas the *τ*_1_ of PE2 and PE3 were 0.57, and 0.84 ns, respectively, which is accordance with the PL results. (Figure 3b; Figure S20 and Table S2, Supporting Information).

The hole‐only devices were fabricated with the structure of indium tin oxide (ITO)/PEDOT:PSS/PVSK/HTL/Au to evaluate the hole mobility of HTLs in devices. As shown in the space charge limited current (SCLC) measurements, PPr‐based devices had the highest hole mobility (0.29 × 10^−2^ cm^2^·s^−1^·V^−1^) than PE3‐based (0.74 × 10^−3^ cm^2^·s^−1^·V^−1^), and PE2‐based devices (0.108 × 10^−2^ cm^2^·s^−1^·V^−1^) (Figure 3c). Moreover, the trap‐filled limited voltages of PPr, PE3, and PE2 were calculated to be 0.12, 0.29, and 0.27 V, respectively, which indicated the best passivation from PPr.^[^ ^20^ ^]^ The fitted results in Nyquist plots confirmed the suppression of the non‐radiative recombination process in PPr films (Figure 3d; Table S3, Supporting Information). The PPr obtained the highest non‐radiative recombination resistance (17 544 Ω) among all three HTL‐based devices. Meanwhile, the PPr‐based devices retained the highest conductivity with the smallest charge transport resistance (91 Ω). The Mott–Schottky plots showed that the PPr‐based devices have the highest built‐in potential (*V*_bi_) of 0.96 V (Figure 3e). This enlarged *V*_bi_ greatly boosts the driving force and facilitates the charge separation, leading to the enhanced *V*_oc_.^[^ ^21^ ^]^

**Figure 4:**
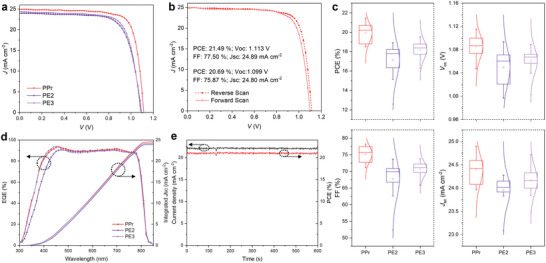
a) *J*–*V* curves of the champion devices based on different HTLs under reverse scan. b) The *J*–*V* curves of the champion PPr device. c) Photovoltaic performance variation for devices based on different HTLs. It displays the mean, with 1.5× outlier range whiskers. d) The EQE spectra of devices based on different HTLs. e) The steady‐state power output of the champion PPr device at the max power point.

To investigate the impact of polymers as HTL in photovoltaics, we fabricated PSCs based on n‐i‐p architecture (ITO/SnO_2_/PVSK/HTL/Au). **Figure** **4**a exhibited the current density‐voltage (*J*–*V*) characteristics curves of PSCs based on different HTL materials. Specifically, the PPr‐based devices achieved the highest PCE of 21.49%, surpassing PE2‐based devices (18.92%) and PE3‐based devices (19.54%), and significantly outperforming the devices based on commercial PEDOT:PSS (16.41%) (Figure 4b,c; Figure S21, Supporting Information). Due to the enhanced hole extraction and modified energy alignment, PPr‐based devices achieved higher *V*_oc_ and *J*_sc_ compared with other HTLs (Figure 4c). The higher *J*_sc_ could be confirmed through external quantum efficiency (EQE) measurements (Figure 4d). The integrated *J*_sc_ was calculated to be 23.93, 24.21, and 24.54 mA cm^−2^ for PE2, PE3, and PPr, respectively, which were consistent with the *J*_sc_ results obtained from the calibrated solar simulator. The performance of PPr device was further assessed via steady‐state power output at the max power point (MPP) (Figure 4e). After continuous MPP tracking for 600 s, the PPr device yielded a stable PCE of 21.04%.

**Figure 5:**
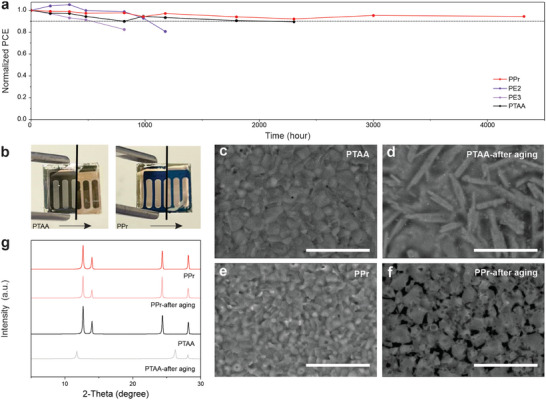
a) Normalized PCE of devices based on different HTLs aged at ambient conditions (25 ± 5 °C and 40 ± 10 RH%). b) The photographs, c–f) the SEM images, and g) XRD patterns of devices based on PTAA and PPr before and after aging two weeks at room temperature in a relative humidity of 75 ± 20%. The scale bar is 5 µm for all samples.

The stability of solar cells is of utmost importance for commercial applications. In addition to PE2 and PE3, we have included PTAA, the widely used HTL material for stability tracking, in our comparative analysis (Figure S22, Supporting Information). To assess humidity stability, we first conducted a device stability test without encapsulation under 40 ± 10 RH%. The resulting data, depicted in **Figures** **5**a and S23 (Supporting Information), reveals that the PCE of PPr‐based devices remained at 94% of its initial efficiency after more than 4000 h of aging, outperforming the performance of PTAA, PE3, and PE2‐based devices. This superior stability can be attributed to its uniform coating and thorough film coverage. To explore the underlying factors for humidity stability, the water contact angle was subsequently measured after allowing the droplet to be sustained on the film for 10 s (Figure S24, Supporting Information). The results demonstrated that the contact angles of PPr (97.4°), PE3 (98.2°), and PE2 (99.2°) were similar, while PTAA had a slightly smaller contact angle (87.2°). Interestingly, yellow circles appeared on the surface of PE3 and PE2‐coated films after 5 min of droplet exposure, which indicates the decomposition of perovskite (Figure S22a, Supporting Information). In contrast, there was no noticeable degradation of perovskite films for PTAA and PPr‐coated films. These results show that PPr has not only high surface hydrophobicity but also high solvent impermeability. To further demonstrate the excellent humidity stability of PPr, the PPr films together with PTAA films were aged in high humidity conditions (75 ± 20%) for 2 weeks. Optical images in Figure 5b exhibit the changes in PTAA and PPr films before and after the aging process. The color of the PTAA film noticeably changed from dark green to light yellow, while the general morphology of PPr films remained unchanged. SEM results showed a significant morphology change in PTAA films (Figure 5c,d). In terms of PPr films, the morphology remained largely unaffected, except for some voids caused by excessive humidity (Figure 5e,f). Additionally, XRD results confirmed the perovskite underwent a phase transformation of perovskite (from *α* to *δ*) in PTAA‐coated films (Figure 5g) while PVSK exhibited superior phase stability for PPr‐coated films. Long‐term light stability and thermal stability tests were also performed to amplify the good stability of PPr‐based devices. As shown in Figure S25 (Supporting Information), the PCE of PPr‐based devices decayed to 80% of the initial value under 1400 h of light soaking. In contrast, the PCE of PTAA‐based devices dropped below 80% only after 300 h. For thermal stability, the PPr‐based devices maintained 76% of the maximum PCE after 1800 h of aging at 65 °C, exceeding the widely used thermally stable PTAA‐based devices (61% of their initial PCE) (Figure S26, Supporting Information).

## 3 Conclusion

In summary, we reported the development of a new pyrrole‐modified dioxythiophene copolymer (PPr) as a promising HTL for PSCs. The incorporation of the pyrrole unit deepened the HOMO and enlarged the bandgap of ProDOT, resulting in better energy alignment. Furthermore, our results indicated that the electron‐rich pyrrole unit could facilitate the formation of Lewis base acid interaction with perovskite, suppressing the defects on the surface. The planar structure of oxidized PPr could also form a robust connection with the perovskite layer, which, with its high hydrophobicity, endowed the structure with outstanding humidity stability. As a result, an optimized PCE of 21.49% was achieved in PPr‐based devices. The PPr devices exhibited excellent stability with 94% of their initial PCEs retained even after being stored in air without any encapsulation for over 4000 h. To the best of our knowledge, this work reports the first‐ever use of ProDOT‐based copolymers as a hole transport layer for perovskite solar cells, which resulted in one of the highest PCE of PSCs with PEDOT derivatives as HTL to date.

## Conflict of Interest

J.M. is a cofounder of Ambilight.

## Supporting information



Supporting Information

## Data Availability

The data that support the findings of this study are available from the corresponding author upon reasonable request.
