# Supplementary material for: A Pyrrole Modified 3,4‐Propylenedioxythiophene Conjugated Polymer as Hole Transport Layer for Efficient and Stable Perovskite Solar Cells
Source: Small. 2024 Oct 28;21(1):2408440. doi: 10.1002/smll.202408440 (PMC11707567; doi:10.1002/smll.202408440)
Supplement: Supplementary file 1 — Supporting Information [file SMLL-21-2408440-s001.docx]

Supplementary Information

A Pyrrole Modified 3,4-propylenedioxythiophene Conjugated Polymer as Hole Transport Materials for Efficient and Stable Perovskite Solar Cells

Yuanhao Tang^1^, Ke Ma^1,2^, Wenhao Shao^1^, Yoon Ho Lee^1.3^, Ashkan Abtahi^3^, Jiaonan Sun^1^, Hanjun Yang^1,3^, Aidan H. Coffey^4^, Harindi Atapattu^5^, Mustafa Ahmed^3^, Qixuan Hu^1^, Wenzhan Xu^1^, Raunak Dani^1^, Limei Wang^1^, Chenhui Zhu^4^, Kenneth R. Graham^5^, Jianguo Mei^3^*, Letian Dou^1,3,6^*

^1^Davidson School of Chemical Engineering, Purdue University; West Lafayette, IN 47907, USA

^2^Global Institute of Future Technology, Shanghai Jiao Tong University; Shanghai, 200240, China

^3^Department of Chemistry, Purdue University; West Lafayette, IN 47907, USA

^4^Advanced Light Source, Lawrence Berkeley National Laboratory, Berkeley, CA 94720, USA

^5^Department of Chemistry, University of Kentucky; Lexington, KY 40506, USA

^6^Birck Nanotechnology Center, Purdue University; West Lafayette, IN 47907, USA

*Corresponding author. Email: dou10@purdue.edu; jgmei@purdue.edu

**Experimental Section**

**Synthesis of PPr**

Figure S1. The failed efforts to make PPr.


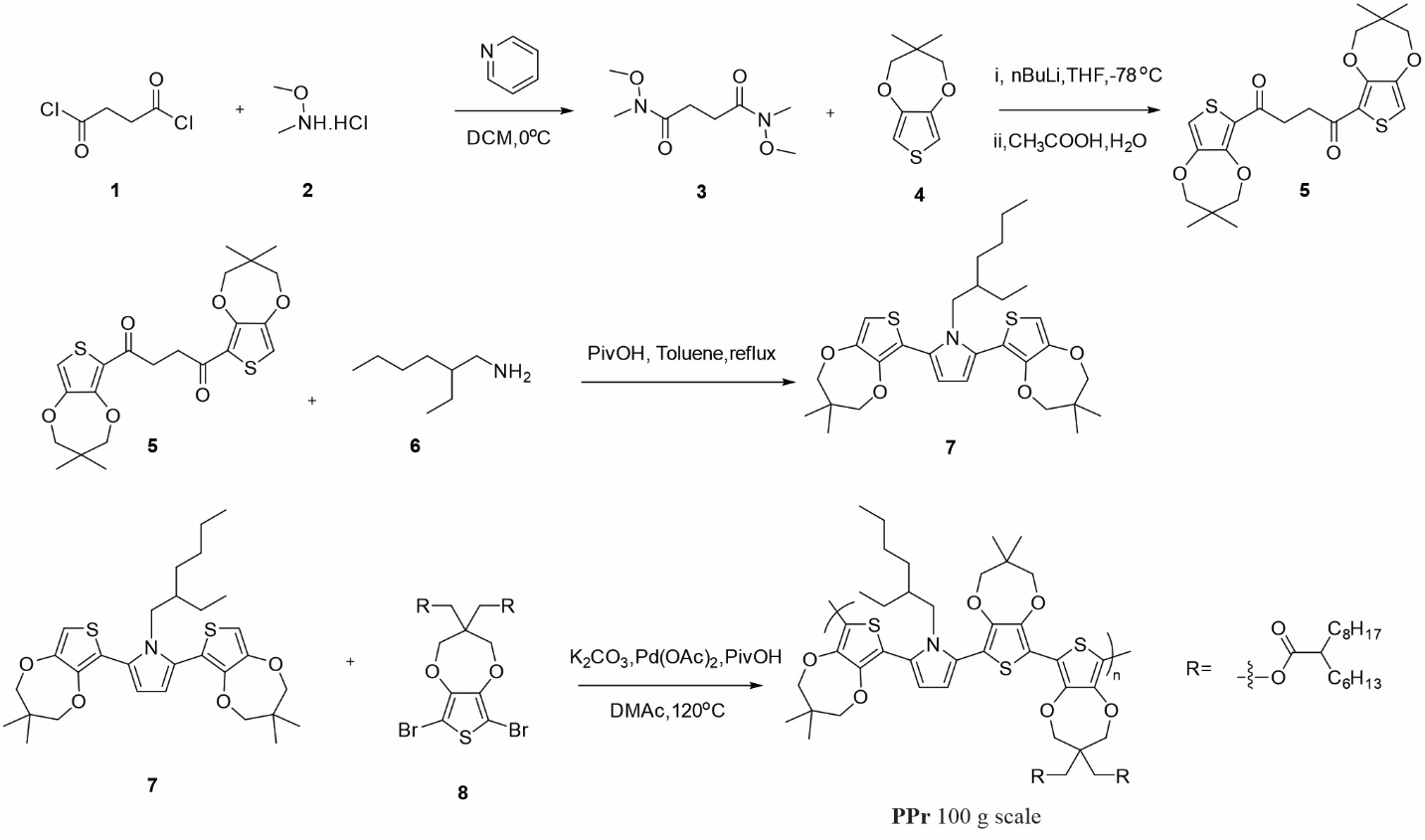
Figure S2. Synthetic route of PPr.

Succinyl chloride (100 g, 645 mmol, 1 eq), compound 2 (144.8 g, 1.48mol, 2.3 eq) were added into a 2L flask with an overhead stir. 1L of DCM is added into the flask and cooled to -10 - 0^o^C. Pyridine (235 g, 2.97mol, 4.6eq) was added into the flask with an addition funnel in 1-2 hours. Then warmed to 20^o^C and stirred for 16 hours. 400 mL water was added into a flask to wash the DCM solution. Then rinsed with 300 mL 6% NaOH solution twice and brine 300 mL for once. The DCM layer was collected and it was removed about 90% of the solvent under vacuum. Hexane 300 mL was added to slurry the product to get pure compound 3 as an off-white solid 81g (61%).^1^H NMR (300 MHz, CDCl_3_), *δ* (ppm): 3.74 (s, 6H), 3.18 (s, 6H), 2.77 (s, 4H).

Compound 4 (96.2 g, 52.2 mmol, 2.6 eq) was added into a 2L flask with a stir bar. After switching the air in the flask with nitrogen anhydrous THF 350 mL was added into the flask to dissolve the solid. The solution was cooled to -78^o^C with an acetone dry ice bath. 200 mL n-BuLi (2.5 M in hexane) (502 mmol, 2.5eq) solution was added into the flask with a tunnel in one hour. The solution slowly warmed to 0^o^C and reacted for 30 mins then cooled to -78^o^C again. N1, N4-dimethoxy-N1, N4-dimethylsuccinamide (41 g, 201 mmol, 1.0 eq) were dissolved in dry THF 300 mL then added into the flask in 30-50 mins at -78^o^C. The mixture was stirred at -78^o^C for 2 hours and then warmed to room temperature (lots of solid precipitates). The reaction was quenched with 40 mL of acetic acid. Then 150 mL water and 650 mL MeOH were added into the flask with a stir. The slurry was filtered and dry to get an off-white solid product of 84 g. The solid dissolved into 504 mL DCM at 40^o^C. Then methanol 1000 mL was added to the DCM solution to precipitate the solid. The slurry was cooled to 0^o^C then filtered and dried to get the diketone product 72 g as a white solid. (yield~80%). ^1^H NMR (300 MHz, CDCl_3_), *δ* (ppm): 6.80 (s, 2H), 3.98 (s, 4H), 3.79 (s, 4H) 3.31 (s, 4H), 1.07 (s, 12H).

Compound 5 (52 g, 115 mmol, 1.0 eq), compound 6 (44.75 g, 346 mmol, 3 eq), Pivalic acid (3.54 g, 34.6 mmol, 0.3 eq), toluene 300 mL were added into a 1L flask equipped with dean stark trap. After switching the air with nitrogen, the flask was heated to reflux for 16 hours under nitrogen. About 4-5 mL water would be collected by Dean-Stark trap. The reaction was cool to 20^o^C under nitrogen. 200 mL water was added to wash the toluene solution twice. The organic layer was collected and distilled into the solvent under vacuum. The residue oil was dissolved into 150 mL hexane and filtered through a silica gel pad (250 g silica gel), washed the silica gel pad with 2 L hexane/DCM(2/1) solution. After removing the solvent, compound 7 was got as a light-yellow sticky oil. 58.4g (yield 93%). ^1^H NMR (300 MHz, CDCl_3_), *δ* (ppm): 6.48 (s, 2H), 6.25 (s, 2H), 3.96 (t, 2H, *J* = 6.0 Hz), 3.78 (s, 8H), 1.37-1.27 (m, 1H), 1.12-0.88 (m, 20H), 0.79 (t, 3H, *J* = 6.0 Hz), 0.60 (t, 3H, *J* = 6.0 Hz).

To a 2 L flask with overhead stir was added compound 7 (58.3 g, 107.2 mmol, 1 eq), compound 8 (91.22 g, 107.2 mmol, 1 eq), pivalic acid (3.28 g, 32.16 mmol, 0.3 eq), K_2_CO_3_ (38.52 g, 278.75 mmol, 2.6 eq), Pd(OAc)_2_ (481.4 mg, 2.14 mmol, 0.02 eq). Vacuum and refill with nitrogen three times. DMAc 570 mL was added into the flask and bubbled with nitrogen for 15 mins. Heated the mixture to 120^o^C for 16 hours. Toluene 600ml was added followed by 600 mL water. Wash the solution at 80^o^C then release out the water phase. 300 mL water is added to wash the solution. After removing the water phase, the polymer solution was added to 3 L acetone with an overhead stir. Filter and dry to get 136 g polymer PPr. (yield~100%).

Figure S3. ^1^H NMR spectrum of compound **3**.

Figure S4. ^1^H NMR spectrum of compound **5**.

Figure S5. ^1^H NMR spectrum of compound **7**.

Figure S6 ^1^H NMR spectrum of PPr. ^1^H NMR (300 MHz, CDCl_3_), *δ* (ppm): 6.30 (s, 2H), 4.30-3.80 (m, 18H), 2.40-2.34 (m, 2H), 1.61-0.65 (m, 89H).


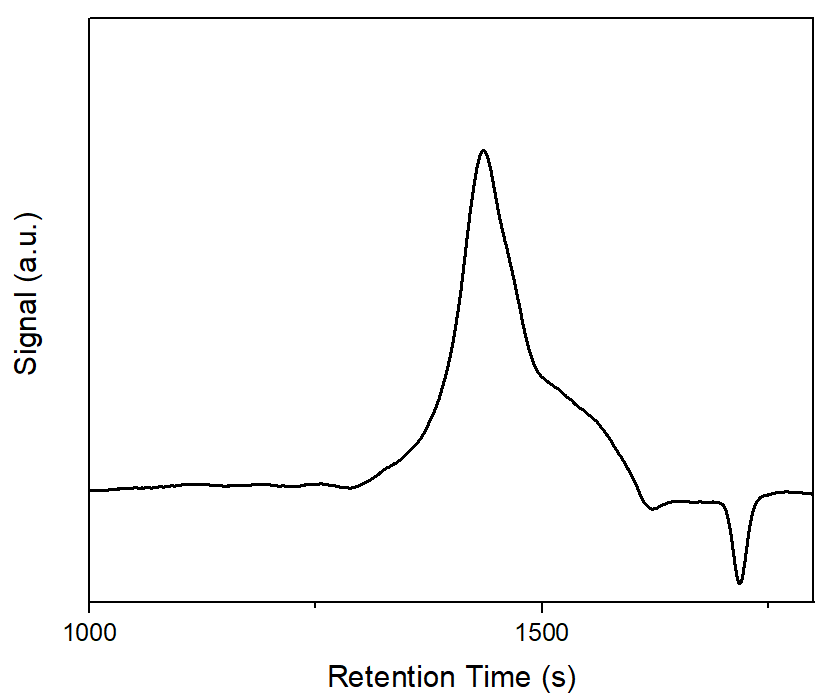


Figure S7. GPC curve of PPr. GPC (THF as solvent) Mn: 1.5k, Mw: 2.7k, PDI 1.76

**Chemicals and Reagents**

PbI_2_ (perovskite grade) and SnO_2_ colloidal solution were purchased from Alfa Aesar. TPFB, CsI, KOH, dimethylformamide (DMF), dimethyl sulfoxide (DMSO), chloroform (CF), chlorobenzene (CB), and isopropyl alcohol (IPA) were purchased from Sigma Aldrich. Poly(triaylamine) (PTAA) was purchased from Ossila. FAI, MACl, and MAI were purchased from GreatCell Solar. Gold pellets were purchased from Kurt J. Lesker. The other chemicals used to synthesize PPr were purchased from commercial companies. All chemicals were directly used without any further purification.

**Characterization**

The Fourier transform infrared spectroscopy (FTIR) measurements were performed on Thermo Nicolet Nexus FT-IR. The thermogravimetric analysis (TGA) and differential scanning calorimetry (DSC) measurements were conducted by SDT-Q600 Simultaneous TGA/DSC. The UV-vis absorption spectra were obtained from the Cary 5000 UV-Vis-NIR Spectrophotometer. Ultraviolet photoelectron spectra (UPS) were acquired using a H Lyman-α photon source (E-LUXTM 121) with a 5 V negative bias. The photon energy used was 10.2 eV and the pass energy was 5.85 eV.

The power X-ray diffraction (XRD) patterns were measured by Rigaku SmartLab with the PhotonMax high-flux 9 kW rotating anode X-ray source (Cu Kα, λ =1.54178 Å), in-plane arm (5-axis goniometer), and HyPix-3000 high energy resolution 2D HPAD detector. The collection of grazing incident wide-angle X-ray scattering (GIWAXS) spectra was carried out at beamline 7.3.3 of the Advanced Light Source located at Lawrence Berkeley National Lab. These measurements utilized an incident angle of 0.12° and wavelength of 1.24 Å (10 keV). The data integration was performed using the Igor Pro 9.01 with the NIKA SAS 2D package.

The scanning electron microscopy (SEM) images were taken using Hitachi S-4800 SEM at 5.0 kV with a secondary electron detector. The cross-section SEM images were obtained with the same equipment as SEM with a different voltage of 2.0 kV. The Asylum Research Cypher ES Environmental AFM was utilized to obtain AFM images in the air. Conductive ITO/glass substrates were prepared for the AFM measurements, and Ti/Pt coated AC240TM-R3 tips from Oxford Instruments were used for the measurements. The AFM measurements were performed in the dark. Photoluminescence spectra (PL) were acquired on a SpectraPro HRS-300. Time-resolved PL (TRPL) was measured based on a home-built PL microscopy using a 40× objective with a numerical aperture of 0.6. A femtosecond pulsed diode laser (Pico-Quant, LDH-P-C-450B) with a wavelength at 447 nm was used as an excitation light source. The emission light was collected by a single-photon avalanche diode and a single-photon counting module (PicoQuant) with 16 ps resolution. The resulting TRPL traces are fitted by a biexponential decay convoluted with instrument response. The fit equation is shown below:

$$y=A_{1}\times e^{\frac{-x}{\tau_{1}}}+A_{2}\times e^{\frac{-x}{\tau_{2}}}$$

The contact angles were analyzed with ImageJ software using the Ramé-hart Model 200. The electricity conductivity of doped HTL films was measured using a facile device. Different polymers (dissolved in chloroform) were spin-coated on a cleaned silicon wafer dynamically at a speed of 3000 rpm. Then the gold is thermally evaporated with a designed mask. The width and length of the non-conducting channel were 0.01 cm and 0.1 cm, respectively. The current-voltage characteristics were measured by Signatone Probe Station (Keithley 4200, 2400 Source meter Linkam hot stage). The electrical conductivity was calculated based on the equation below:

$$\sigma=\frac{L}{Wd}\times\frac{dV}{dI}$$

where *V* is the measured voltage, *I* is the measured current, *L* = 0.1 cm is the length of the channel. *W* = 0.01 cm is the width of the channel, and d ≈ 60 nm is the thickness of the HTL.

The hole mobility of doped HTL films was measured based on holy-only devices with an architecture of ITO/PEDOT:PSS/HTL/Au via space-charge-limited current (SCLC) measurement. The filtered PEDOT:PSS solution was spin-coated dynamically on cleaned ITO substrates at a speed of 5000 rpm, followed by annealing at 170 ℃ for 20 mins. The HTLs, and gold were then spin-coated, and evaporated, respectively, using the same procedure mentioned above. The Mott-Gurney law was used to fit the curve, as shown below:

$$J=\frac{9\mu\varepsilon_{0}\varepsilon_{r}V^{2}}{8L^{3}}$$

where *J* is the current density, *L* is the thickness of the active layer, *μ* is the hole mobility, *ε*_r_ ≈ 3 is the relative dielectric constant of the polymer, *ε*_0_ is the permittivity of free space, and V is the voltage of the device.

The J-V characteristics measurement were carried out as follows: J-V curves were recorded in a N2 glove box using a solar simulator (Enlitech SS-F5-3A) with 1 sun intensity and AM 1.5G irradiation from an Xe lamp. The light intensity of 100 mW cm-2 was calibrated before each use with a standard Si reference cell certified by NREL, and the active area of each device was measured with an Olympus microscope. The voltage sweep range was from 1.2 V to -0.2 V (reverse) and from -0.2 V to 1.2 V (forward), with an interval of 0.02 V. EQE measurements were performed at zero bias in air on a home-built setup equipped with a preamplifier and a lock-in amplifier with a 161 Hz chopper frequency. The light source was also calibrated with a Si diode (818-UV-L). The I-t curves at the voltage of highest power were tracked for maximum power point (MPP) measurements.

The hole mobilities of devices based on different HTLs were determined by conducting SCLC measurements for hole-transport-only devices in the dark, with a configuration of ITO/PEDOT:PSS/perovskite/HTLs. The data was fitted followed by the same procedure above. The voltage range was 0 V to 5 V with a 20-mV step size. For the MS and EIS measurements, impedance spectra were recorded in the frequency range of x Hz to X MHz using a X. The measurements were performed under dark conditions, and a sinusoidal voltage signal with an amplitude of X mV was applied. The trap-filled limited voltage was obtained by extending the line segment of the trap-filled limited region to intersect the x-axis.

The moisture stability of perovskite devices was evaluated by placing unencapsulated devices in an ambient environment. The thermal stability of unencapsulated perovskite devices was tested by placing the devices directly onto heating plates in a glove box. The light stability of unencapsulated perovskite devices was conducted by placing the devices under 1 sun illumination in the glovebox.

**Calculation Methods.**

Geometry optimizations for PPr, PE2, and PE3 were carried out by means of Density Function Theory (DFT) as implemented in the Gaussian 16 package^[1]^ using the B3LYP^[2]^ functional and 6-311G(d,p)^[3]^ basis set in a vacuum. Surface electrostatic potentials were calculated with the multiwfn^[4]^ software and visualized in the VMD^[5]^ software according to the procedure previously reported.^[6]^ Simplified methyl-terminated monomer structures were used to represent polymeric PPr, PE2, and PE3. Long side chains were reduced to prevent complexity except for that attached to the pyrrole unit in PPr, which was left adequately bulky to induce comparable steric hindrance.

**Perovskite Photovoltaic Device Fabrication.**

The ITO substrates were cleaned in sequence with soap water, deionized water (DIW), acetone, and isopropyl alcohol (IPA) in an ultrasonic cleaner. The SnO_2_ as electron transport layer suspension solution was diluted with DIW and IPA, and then spin-coated on the ITO substrates, followed by annealing at 150℃ for 30 mins. After 15 mins of UV-ozone treatment, the SnO_2_ layer was passivated by 10 mm KOH solution, followed by annealing at 150℃ for another 30 mins. The perovskite layer was spin-coated via 2 steps method. 1.5 M PbI_2_ with 0.075 M CsI were dissolved in DMF/DMSO (v/v, 9/1) and then spin-coated at 1500 rpm for 30 s. After annealing at 70℃ for 1 min, the mixture solution of FAI/MACl/MAI (0.52:0.15:0.03 M) in IPA was spin-coated at 1800 rpm for 30 s, followed by annealing 70 ℃ for 1 min in glovebox and 150℃ for 15 mins in air. After that, Cl4Tm was used to passivate the perovskite layer by spin-coating at 4000 rpm for 30 s and annealing at 100℃ for 10 mins in the glovebox. For the hole transport layer, PPr (10 mg/mL in chloroform), PE3 (10 mg/mL in chloroform), PE2 (10 mg/mL in chloroform), or PTAA (18 mg/mL in chlorobenzene) was doped with TPFB (15 wt.%) and stirred overnight under light soaking. HTLs were then dynamically spin-coated at 3000 rpm for 30 s, followed by annealing at 80℃ for 5 mins. Finally, a 90 nm Au layer was deposited by a thermal evaporator. The active area of the electrode was 0.05 cm^2^.

**Statistical Analysis**

The statistical results in the figures display the mean, with 1.5× outlier range whiskers.

**Reference**

[1] M. J. Frisch, G. W. Trucks, H. B. Schlegel, G. E. Scuseria, M. A. Robb, J. R. Cheeseman, G. Scalmani, V. Barone, G. A. Petersson, H. Nakatsuji, X. Li, M. Caricato, A. V. Marenich, J. Bloino, B. G. Janesko, R. Gomperts, B. Mennucci, H. P. Hratchian, J. V. Ortiz, A. F. Izmaylov, J. L. Sonnenberg, Williams, F. Ding, F. Lipparini, F. Egidi, J. Goings, B. Peng, A. Petrone, T. Henderson, D. Ranasinghe, V. G. Zakrzewski, J. Gao, N. Rega, G. Zheng, W. Liang, M. Hada, M. Ehara, K. Toyota, R. Fukuda, J. Hasegawa, M. Ishida, T. Nakajima, Y. Honda, O. Kitao, H. Nakai, T. Vreven, K. Throssell, J. A. Montgomery Jr., J. E. Peralta, F. Ogliaro, M. J. Bearpark, J. J. Heyd, E. N. Brothers, K. N. Kudin, V. N. Staroverov, T. A. Keith, R. Kobayashi, J. Normand, K. Raghavachari, A. P. Rendell, J. C. Burant, S. S. Iyengar, J. Tomasi, M. Cossi, J. M. Millam, M. Klene, C. Adamo, R. Cammi, J. W. Ochterski, R. L. Martin, K. Morokuma, O. Farkas, J. B. Foresman, D. J. Fox, Gaussian 16 Rev. C.01, Wallingford, CT, **2016**.

[2] a)A. D. Becke, *J Chem Phys* **1993**, *98*, 5648-5652; b)P. J. Stephens, F. J. Devlin, C. F. Chabalowski, M. J. Frisch, *J Phys Chem-Us* **1994**, *98*, 11623-11627.

[3] A. D. Mclean, G. S. Chandler, *J Chem Phys* **1980**, *72*, 5639-5648.

[4] T. Lu, F. W. Chen, *J Comput Chem* **2012**, *33*, 580-592.

[5] W. Humphrey, A. Dalke, K. Schulten, *J Mol Graph Model* **1996**, *14*, 33-38.

[6] W. C. Liang, P. L. Luo, Y. P. Lee, *Phys Chem Chem Phys* **2021**, *23*, 11082-11090.


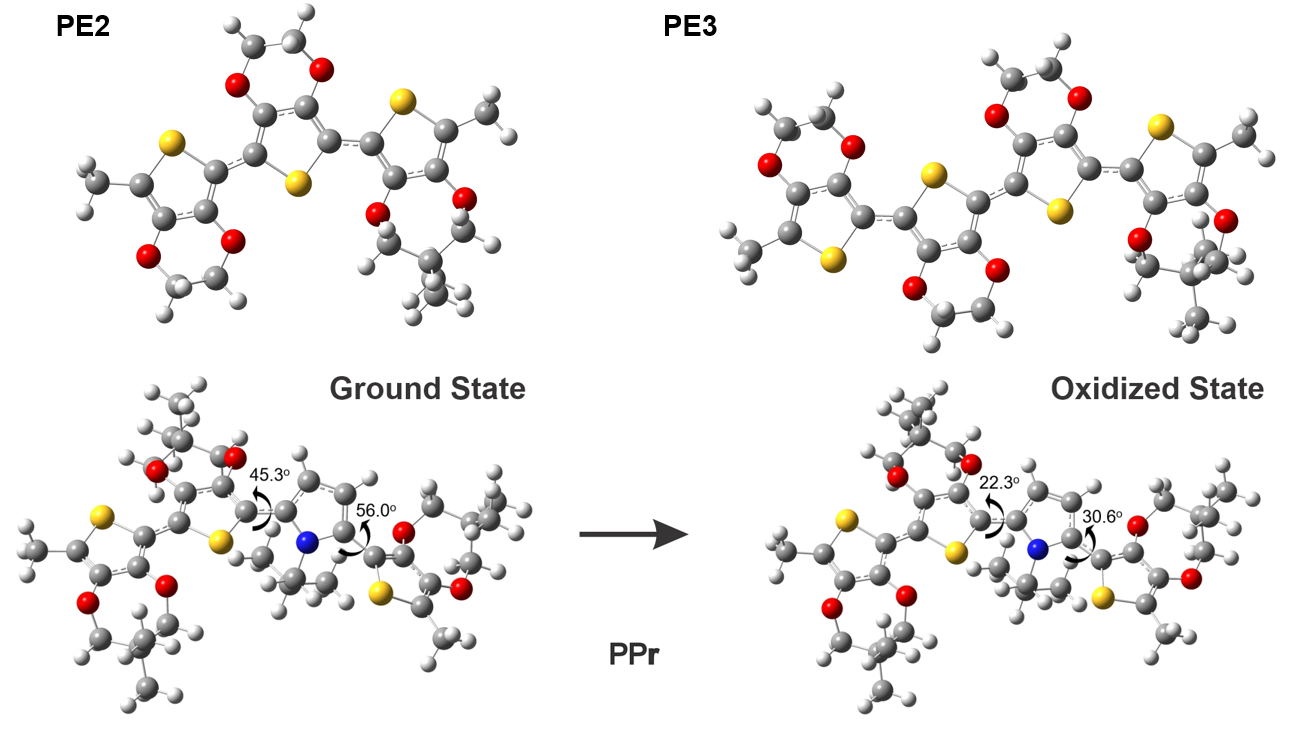


Figure S8. The DFT optimized monomer molecular structure of PE2, PE3, and PPr before and after being oxidized.


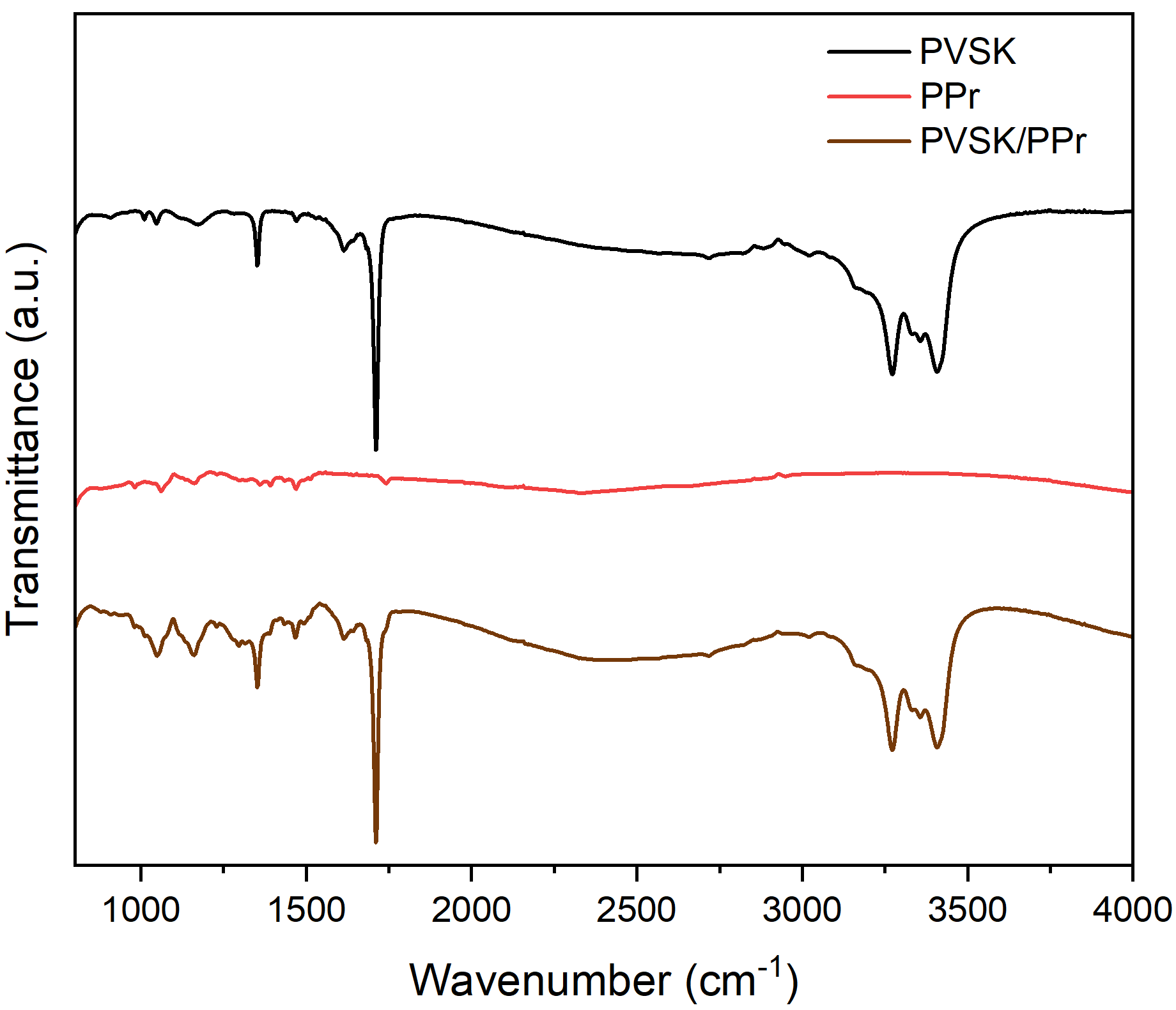


Figure S9. FT-IR spectra of PVSK, PPr, and PVSK/PPr.


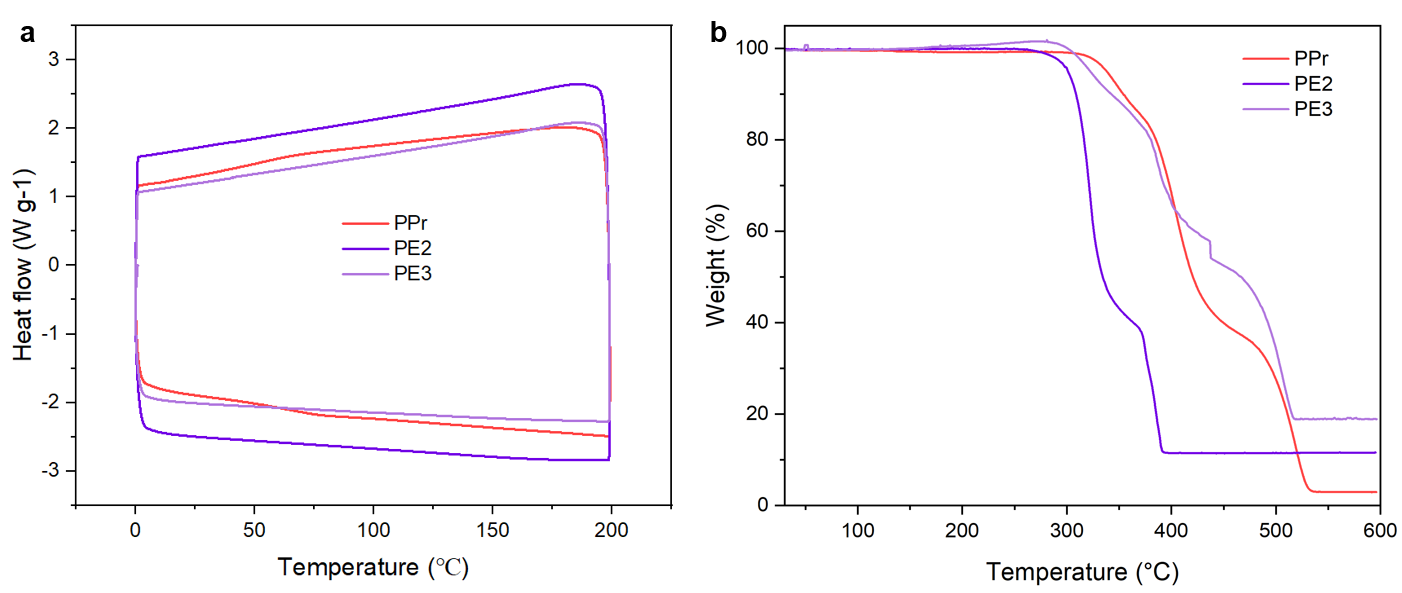


Figure S10. The DSC (a) and TGA (b) spectra of PPr, PE2, and PE3.


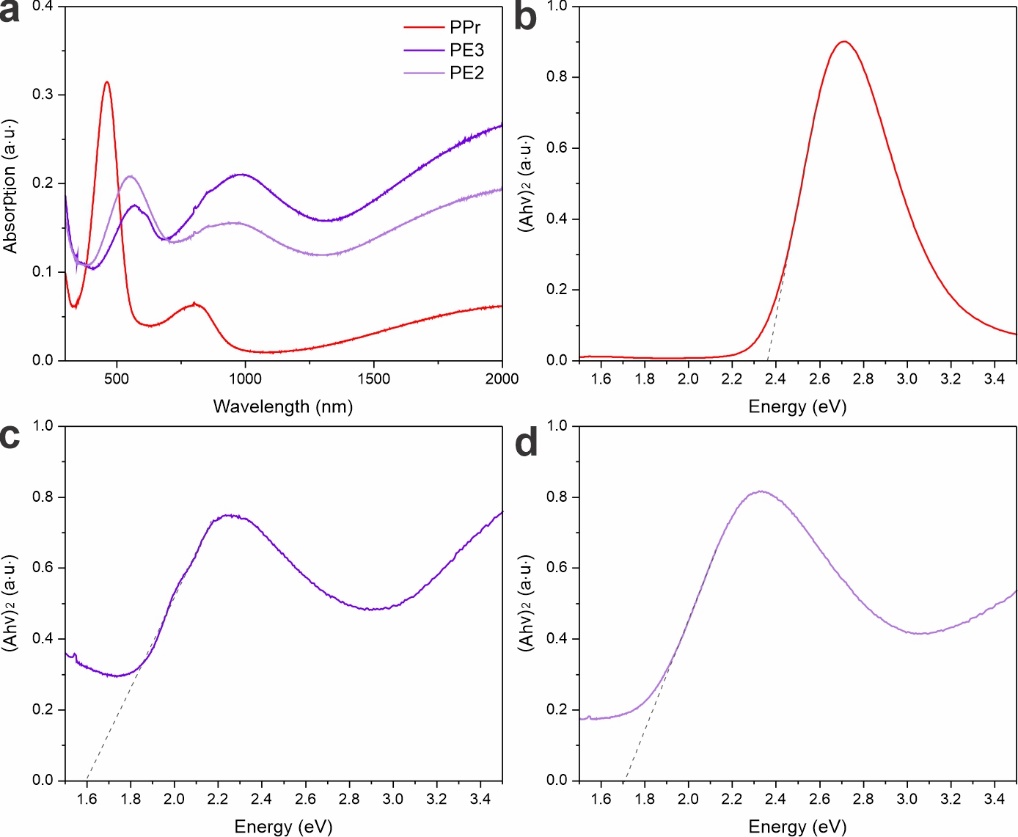


Figure S11. (a) UV-Vis spectra of films based on different HTLs. The Tauc plots of (b) PPr, (c) PE3, and (d) PE2. The optical band gap can be estimated by using equation $\left( h\nu-E_{g} \right)=\left( Ah\nu\right)^{\frac{1}{\gamma}}$ where ν is 0.5 for direct bandgap materials.


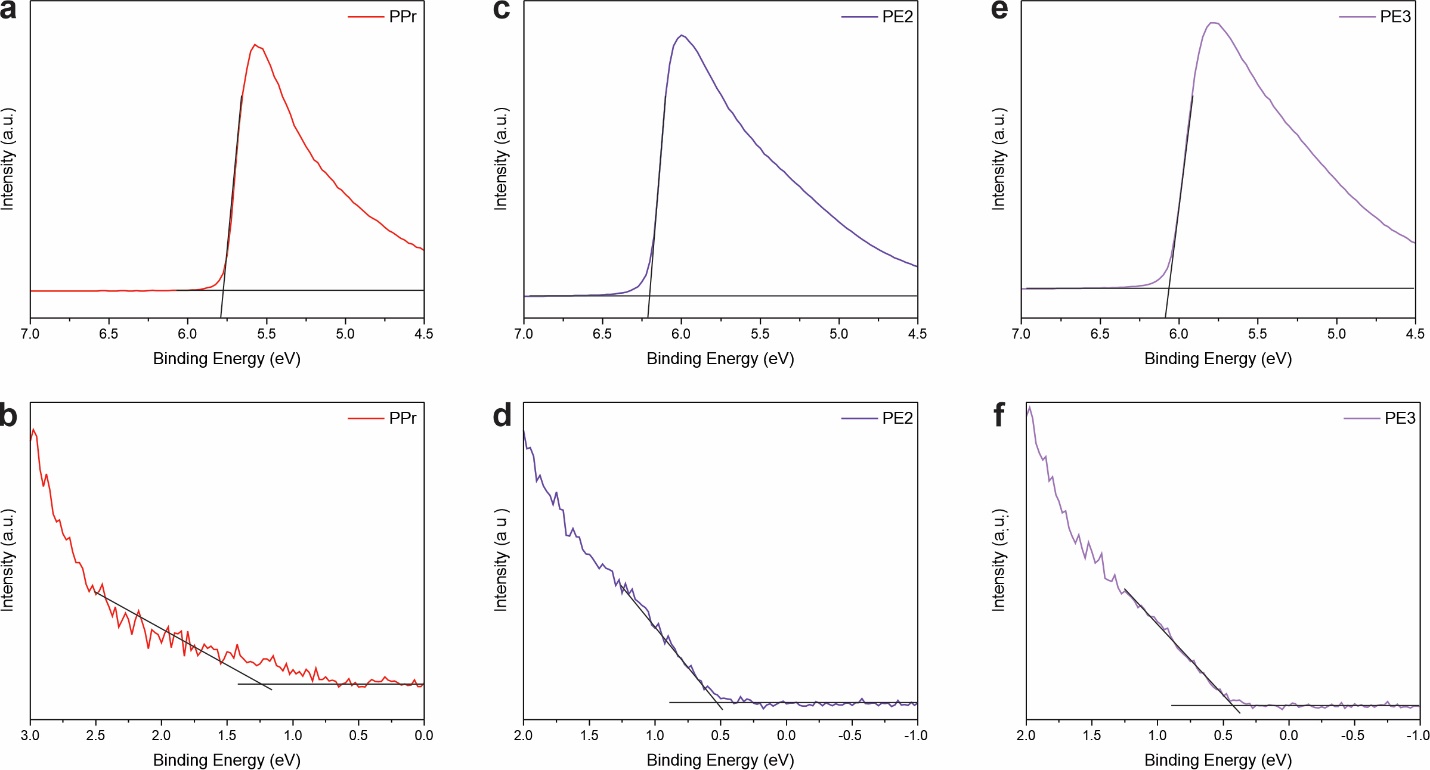


Figure S12. Ultraviolet photoemission spectra secondary electron cut-off regions of perovskite films based on (a) PPr, (c) PE2, and (e) PE3, and valence band onset regions of perovskite films based on (b) PPr, (d) PE2, and (f) PE3.


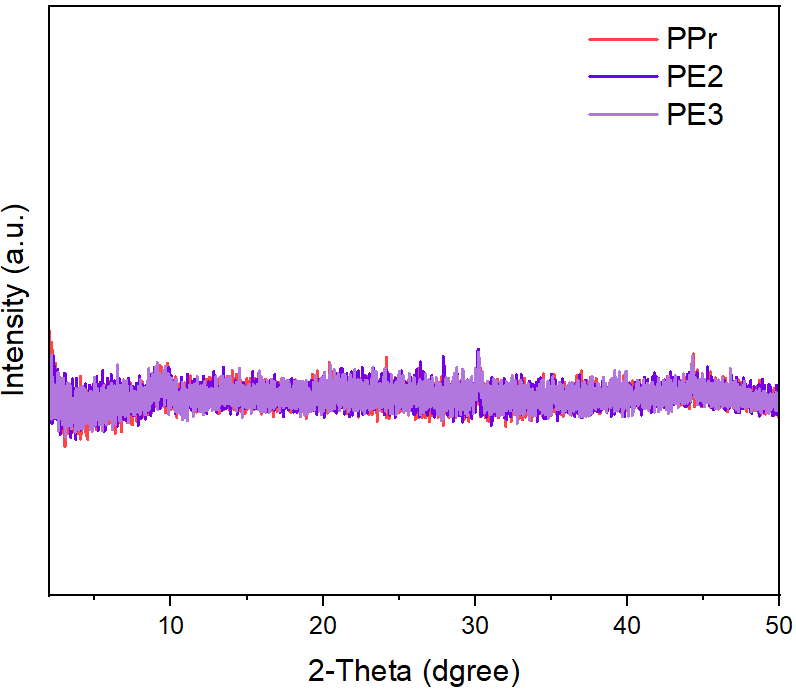


Figure S13. XRD patterns of different HTLs.


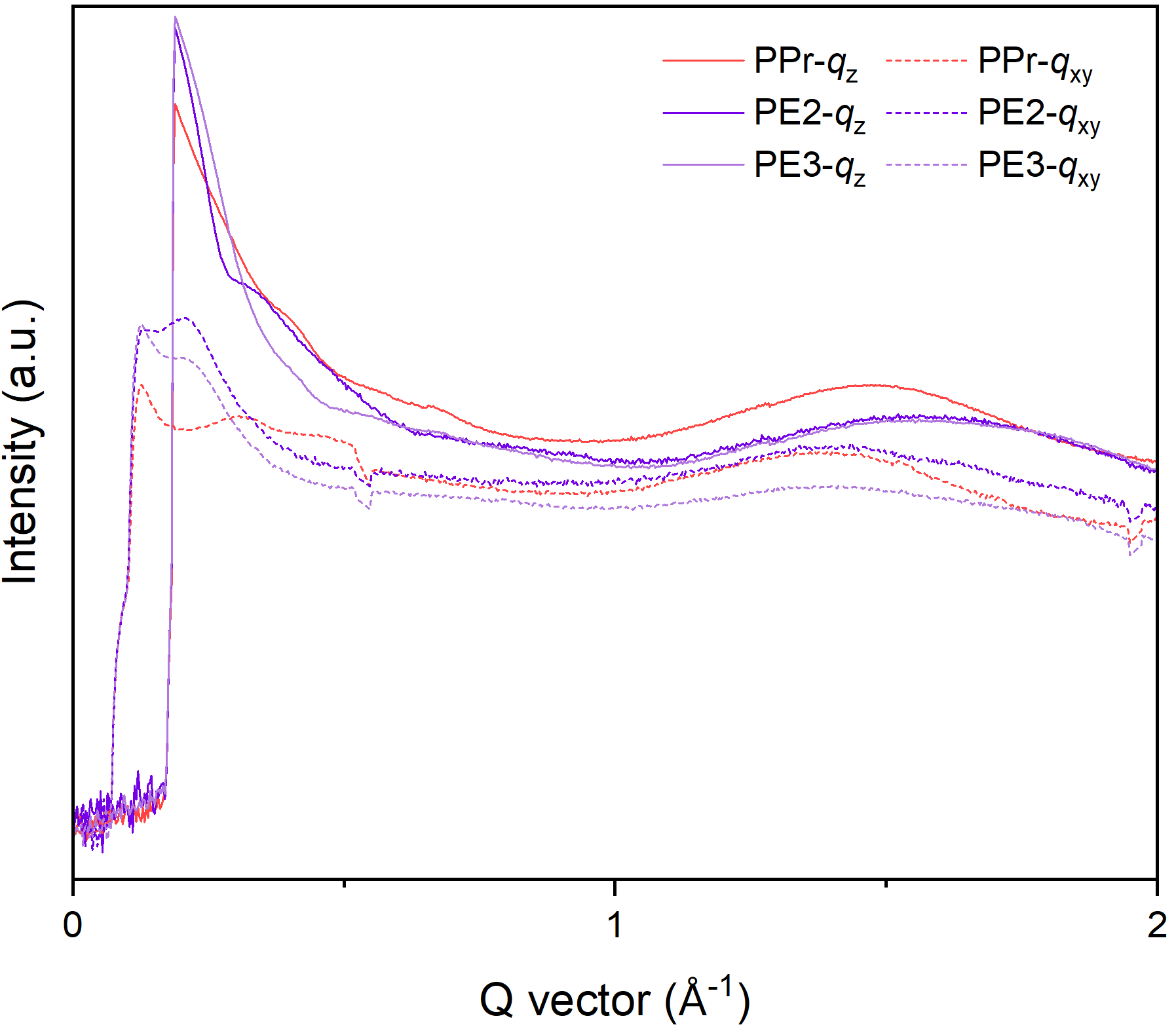


Figure S14. The out-of-plane and in-plane line cuts of PPr, PE2, and PE3 in GIWAX profiles.


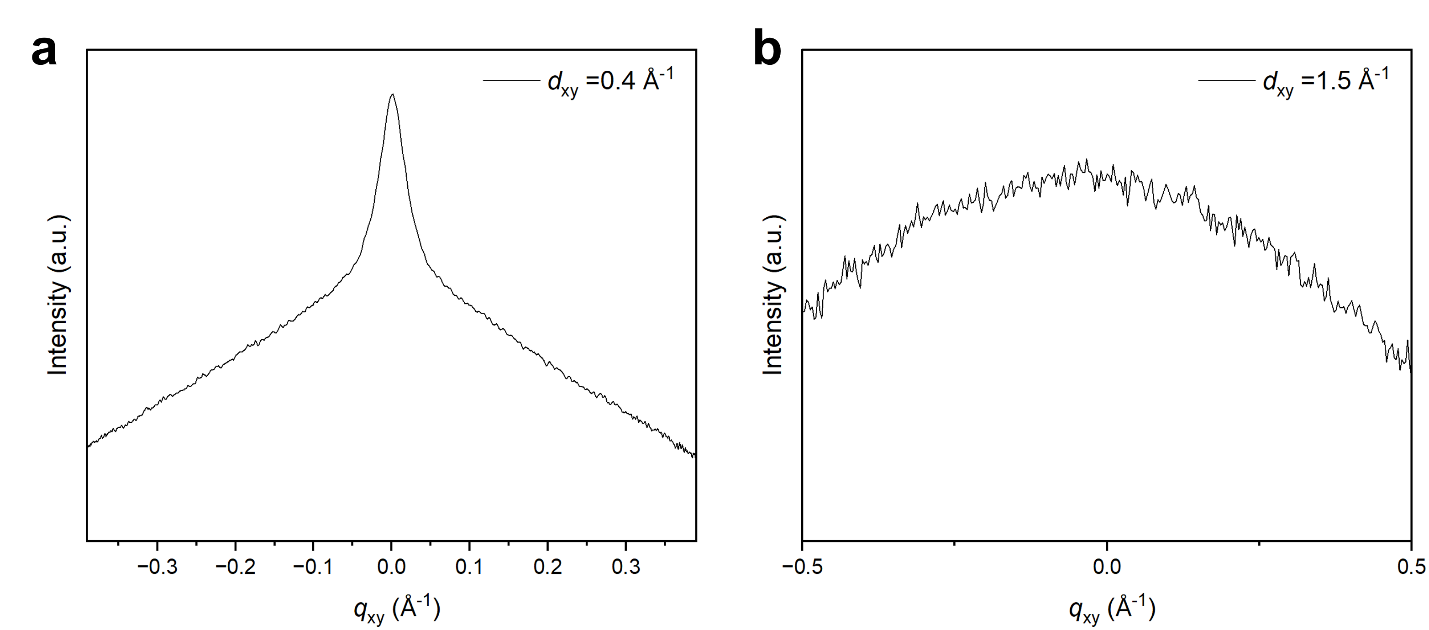


Figure S15. The ellipse line cuts of PPr at *d*_xy_ = 0.4 Å^−1^ (a) and *d*_xy_ = 1.5 Å^−1^ (b) in GIWAX profiles.


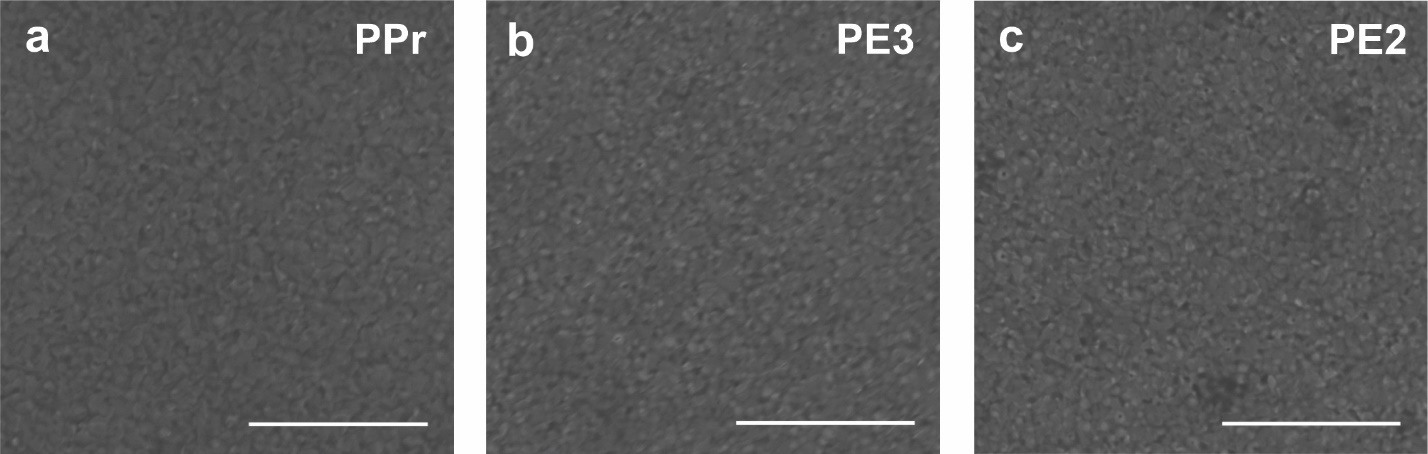


Figure S16. SEM images of films based on (a) PPr, (b) PE3, and (c) PE2. The scale bar is 5 μm for all samples.


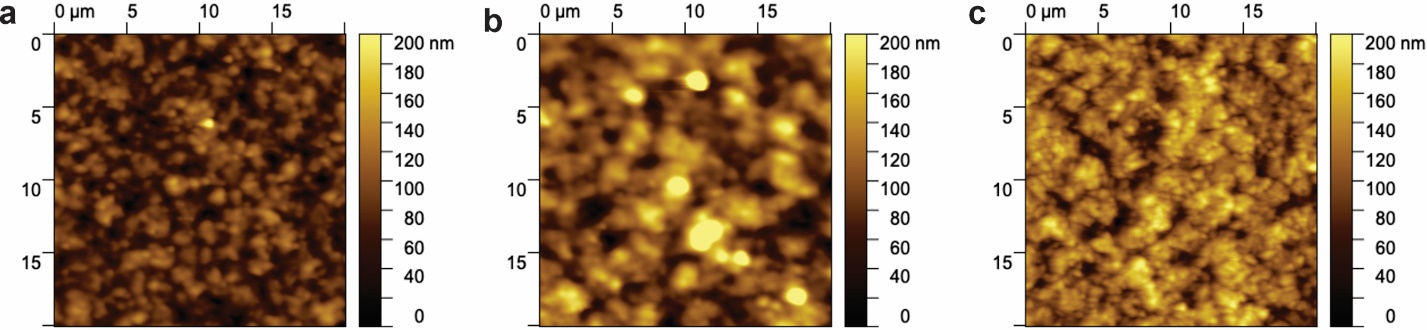


Figure S17. AFM images of films based on (a) PPr, (b) PE3, and (c) PE2.


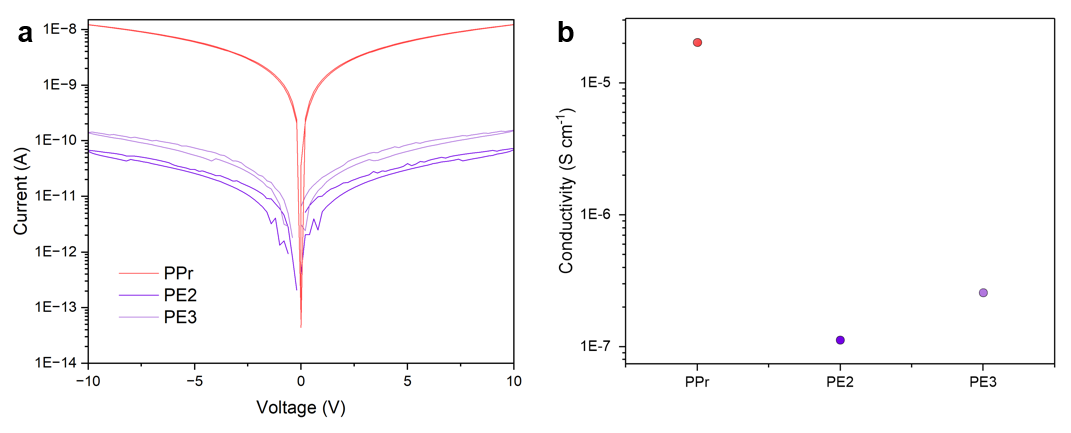


Figure S18. (a) Current-voltage curves, and (b) calculated conductivity of PPr, PE2, and PE3. The *J*-*V* curves and conductivity are measured based on a structure of silicon wafer/polymer/gold.


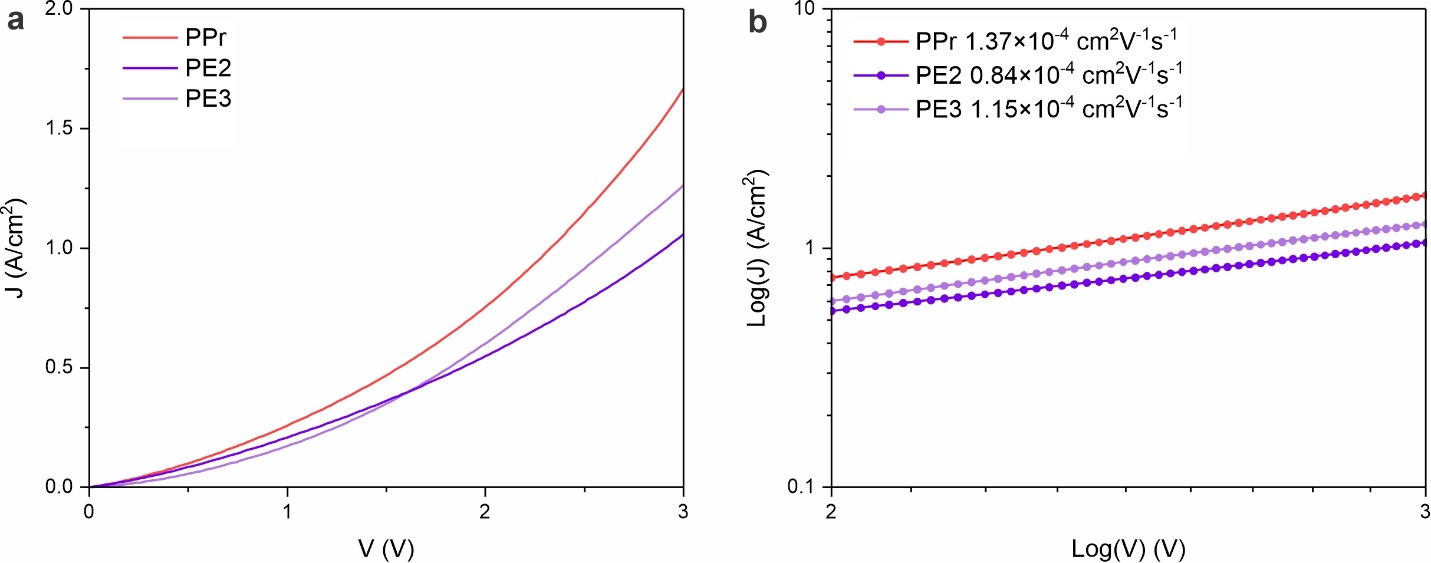


Figure S19. Dark *J*-*V* curves (a) and log(*J*)-log(*V*) curves (b) of PPr, PE2, and PE3. The J-V curves are measured based on hole-only device with a structure of ITO/PEDOT: PSS/polymer/gold.

Figure S20. The enlarged TRPL results of pristine PVSK and PVSK with PPr, PE3, and PE2 films.


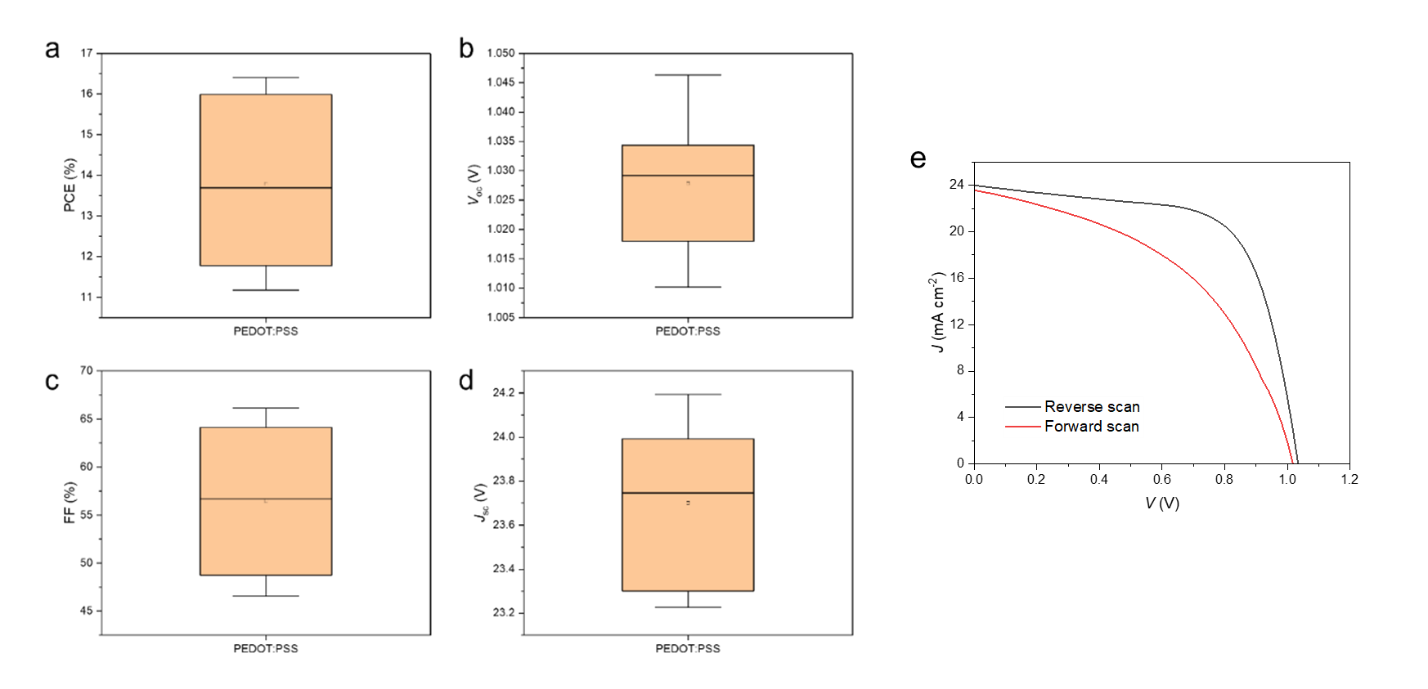


Figure S21. The statistics showing the PCE (a), *V*_oc_ (b), FF (c), *J*_sc_ (d) of PEDOT: PSS-based devices. (e) The *J*-*V* curve of champion PEDOT:PSS-based device. Panels a to d display the mean, with 1.5× outlier range whiskers.


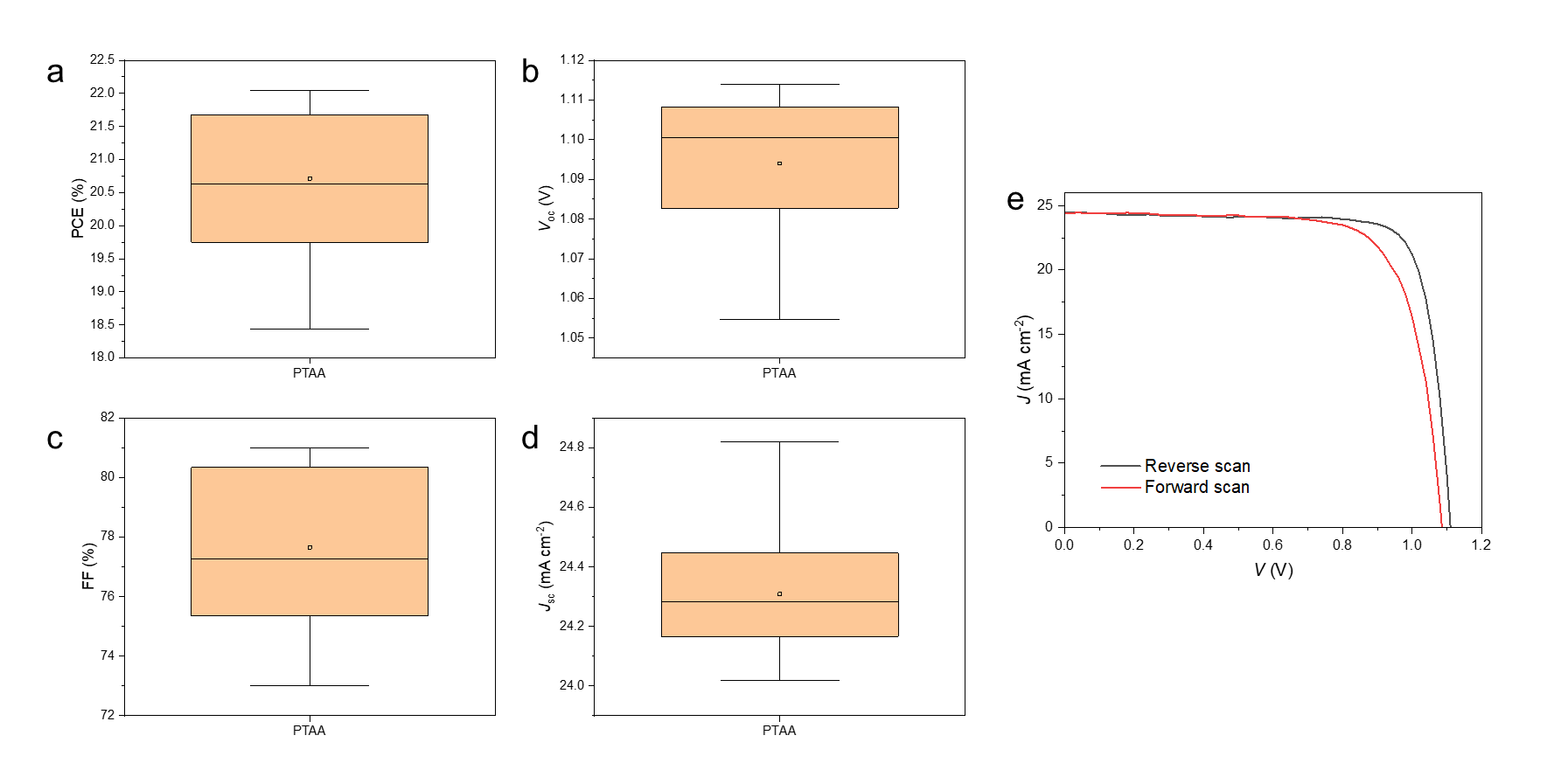


Figure S22. The statistics showing the PCE (a), *V*_oc_ (b), FF (c), *J*_sc_ (d) of PTAA-based devices. (e) The *J*-*V* curve of champion PTAA-based device. Panels a to d display the mean, with 1.5× outlier range whiskers.


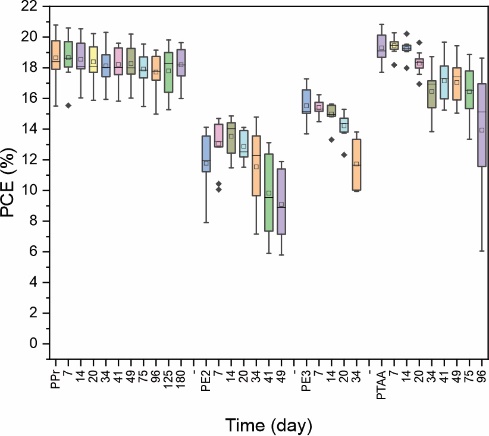


Figure S23. The stability of devices based on PPr, PE2, PE3, and PTAA aged in ambient conditions. It displays the mean, with 1.5× outlier range whiskers.


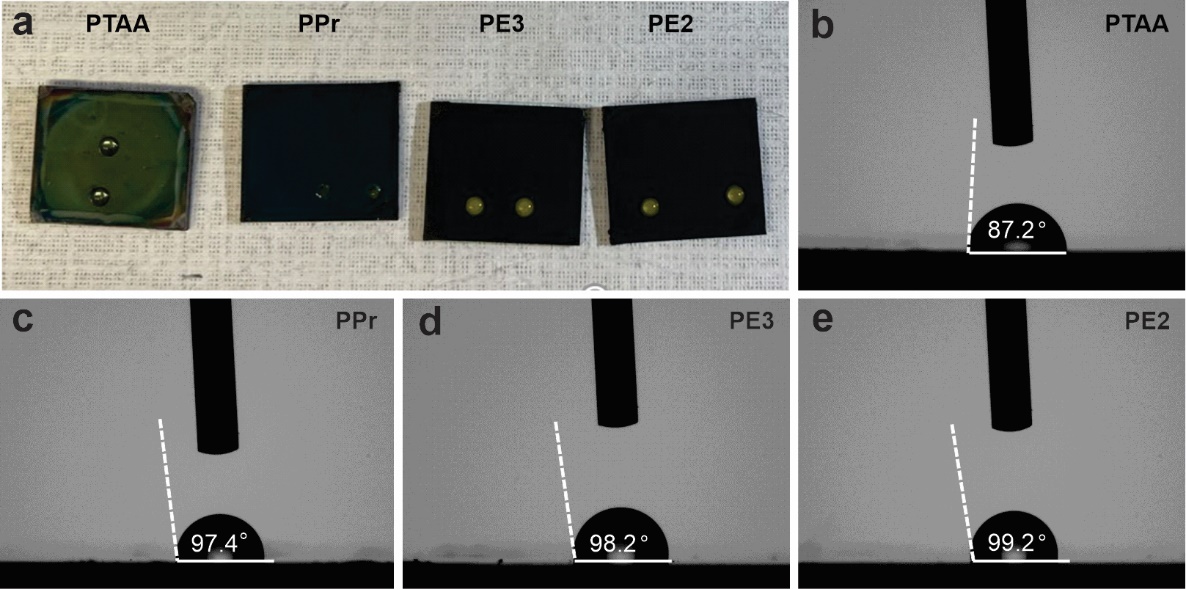


Figure S24. (a) Optical images of films based on different HTLs under water contact measurements. Images of water droplet contact angles on surfaces of (b) PTAA, (c) PPr, (d) PE3, and (e) PE2.


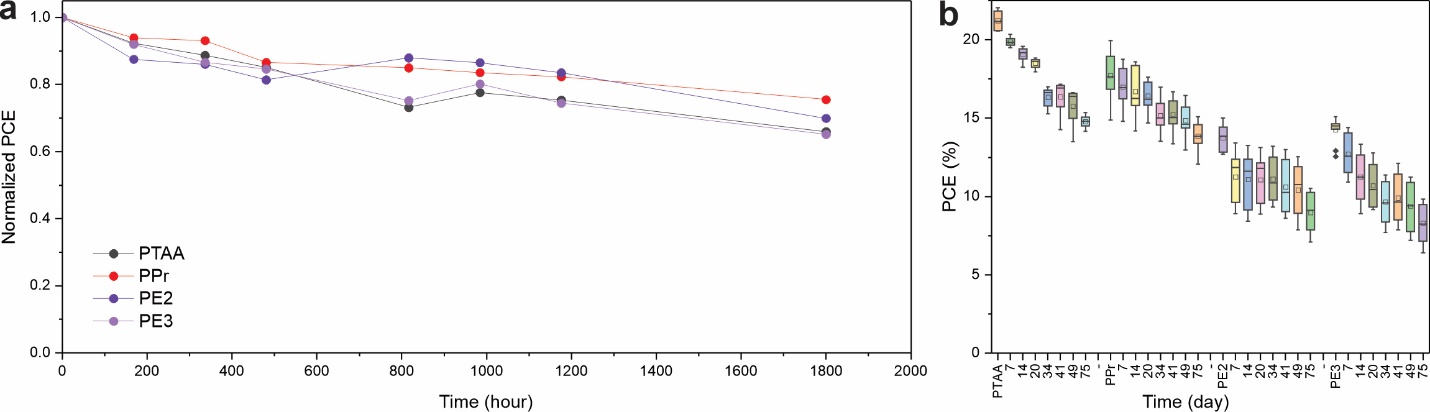


Figure S25. The stability of devices based on PPr, PE2, PE3, and PTAA aged under 0.8 sun light in the glovebox. Panel b displays the mean, with 1.5× outlier range whiskers.


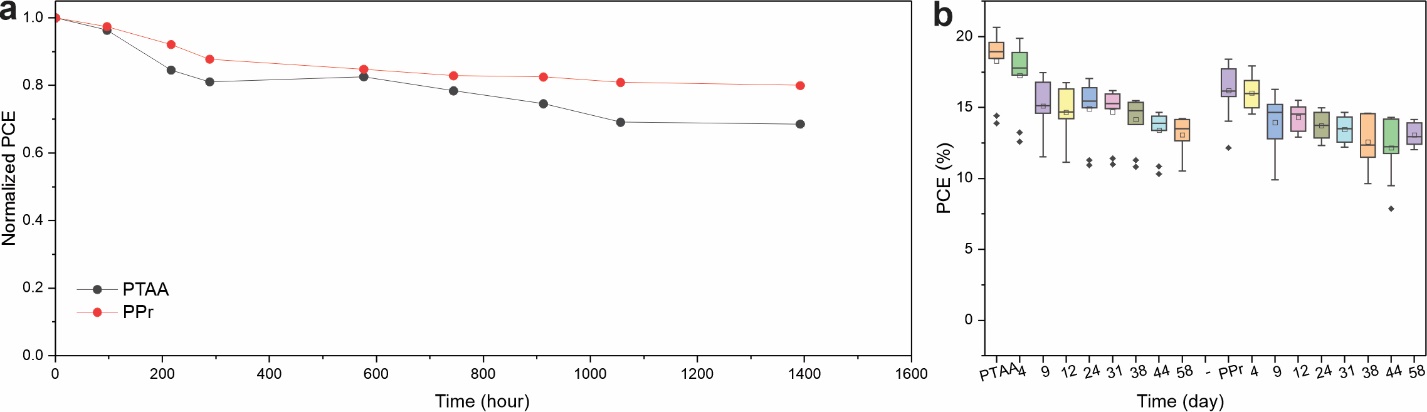


Figure S26. The stability of devices based on PPr, PE2, PE3, and PTAA aged under 65 ℃. Panel b displays the mean, with 1.5× outlier range whiskers.

| **Name** | **LUMO_DFT-monomer_ (eV)** | **HOMO_DFT-monomer_ (eV)** | **Bandgap_UV-Vis_ (eV)** | **Work function (eV)** | **Onset (eV)** | **LUMO_exp_ (eV)** | **HOMO_exp_ (eV)** |
| --- | --- | --- | --- | --- | --- | --- | --- |
| PPr | -1.25 | -4.69 | 2.36 | 4.43 | 0.78 | -2.85 | -5.21 |
| PE2 | -1.26 | -4.50 | 1.71 | 3.97 | 0.53 | -2.79 | -4.50 |
| PE3 | -1.46 | -4.28 | 1.6 | 4.15 | 0.43 | -2.92 | -4.58 |

Table S1 Energy diagram data of PPr, PE2, and PE3.

Table S2 TRPL fitting data of PVSK, PPr, PE2, and PE3.

|  | A_1_ | *τ*_1_ | A_2_ | *τ*_2_ |
| --- | --- | --- | --- | --- |
| PVSK | 0.22 ± 0.01 | 2.06 ± 0.16 | 0.44 ± 0.01 | 59.66 ± 1.34 |
| PPr | 0.64 ± 0.01 | 0.55 ± 0.01 | 0.23 ± 0.01 | 40.64 ± 0.39 |
| PE2 | 0.56 ± 0.01 | 0.57 ± 0.01 | 0.31 ± 0.01 | 89.92 ± 1.71 |
| PE3 | 0.60 ± 0.01 | 0.84 ± 0.01 | 0.33 ± 0.01 | 49.21 ± 0.43 |

Table S3 The fitted Nyquist plots results of PPr, PE2, and PE3.
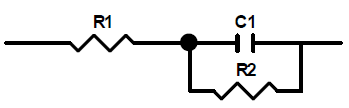


|  | ***R*_1_(*R*_s_)** | ***C*_1_(*C*)** | ***R*_2_(*R*_sh_)** |
| --- | --- | --- | --- |
| PPr | 91.01 | 3.815 × 10^-9^ | 17544 |
| PE2 | 195.9 | 6.219 × 10^-9^ | 2436 |
| PE3 | 124.5 | 6.411 × 10^-9^ | 5235 |
